# Soil Fungal Diversity and Community Structure of *Russula griseocarnosa* from Different Sites

**DOI:** 10.3390/microorganisms13030490

**Published:** 2025-02-22

**Authors:** Zhen Li, Ruoxi Liang, Fei Yu

**Affiliations:** 1 College of Forestry, Shanxi Agricultural University, Jinzhong 030801, China; lizhen@sxau.edu.cn; 2 Honors College, Northwestern Polytechnical University, Xi’an 710129, China; liangruoxi@mail.nwpu.edu.cn

**Keywords:** *Russula griseocarnosa*, mycosphere soil, soil fungi

## Abstract

*Russula griseocarnosa* is an important ectomycorrhizal edible fungus whose economic and nutritional value are both high. To better understand which abiotic and biotic factors affect the growth of *R. griseocarnosa*, this study examined the mycosphere soil of *R. griseocarnosa* growing in five sites. The soil fungal communities of *R. griseocarnosa* from five sites of Fujian, Guangxi, and Yunnan Provinces were sequenced by Illumina MiSeq technology, and their community structure comprehensively analyzed in combination with a suite of soil physicochemical properties. The results revealed significantly greater levels of available potassium (AK), available nitrogen (AN), and available phosphorus (AP) in mycosphere soil than bulk soil, and that *R. griseocarnosa* prefers acidic soil, with *Penicillium*, *Trichoderma*, *Talaromyces*, *Mortierella*, *Tolypocladium*, *Chloridium*, *Oidiodendron*, and *Umbelopsis* being the main dominant fungal taxa. Different geographical sites had different indicator fungal genera, and the similarity of fungal communities in the mycosphere decreased with increasing geographical distance among them. Soil pH was the major abiotic factor influencing the structure of the mycosphere fungal communities. Management strategies such as nitrogen, potassium, phosphorus mixed fertilizer, and fungal fertilizer can promote the conservation and sustainable utilization of *R. griseocarnosa*.

## 1. Introduction

*Russula griseocarnosa* [1] is a kind of wild ectomycorrhizal fungus prized for its edible and medicinal value that has symbiotic relationships with Fagaceae wood plants, but it still cannot be cultivated artificially [2]. Not only does *R. griseocarnosa* contain mineral elements, such as potassium, calcium, and magnesium, but also various active substances, such as protein, amino acid, fatty acid, and polysaccharide, all of which play a potential role in the prevention of anemia and tumors in humans [3]. The current market price of fresh *R. griseocarnosa* fruit bodies is USD 35–45/kg, while the price of their dry products is USD 140–180/kg. Due to extreme climate and excessive hand-picking, the production area has shrunken, resulting in a vicious cycle of diminishing supply and rising prices of *R. griseocarnosa*. The formation of *R. griseocarnosa* fruit bodies is closely related to a warm and humid climate environment [2]. As an ectomycorrhizal fungus, this species is highly affected by its ecological interactions with surrounding biological factors, especially the soil microbiota.

Soil fungi are vital microbial components belowground, and besides their key ecological functions, such as organic matter decomposition and nutrient cycling, also play a crucial role in the growth and development of ectomycorrhizal fungi and the formation of fruiting bodies [4]. Fungi are the main biotic agents that create and maintain soil structure by secreting extracellular compounds and physically binding soil through mycelium networks [5,6]. Work by Yu et al. [4] found that there exist indicator genera of *Russula* sporocarp formation in soil fungi, such as *Clitocybula*, *Phellodon*, and *Tomentella*, in addition to indicator genera capable of coexisting symbiotically with *Russula*, such as *Acremonium* and *Cladophalophora*. Soil fungi in the mycosphere of the edible *Tricholoma matsutake* can promote the material exchange between *T. matsutake* and its host plants, thereby playing a critical role in the fruit body development of *T. matsutake* and the formation of its fruiting bodies [7]. Similarly, *Penicillium* spp. promote the fruit body development of *Lactarius hatsudake* [8]. To improve the yield and the commercial value of *R. griseocarnosa*, its soil fungal community characteristics should be studied to improve their conservation and increase yield.

Many studies have investigated the mycosphere soil bacteria, and other aspects of *R. griseocarnosa* [2], but there is relatively little research on the diversity and structure of the soil fungi dwelling in its mycosphere. Ectomycorrhizal fungi, forming a symbiotic relationship with host plants, interact physically and metabolically with microorganisms in surrounding habitats [9] and select the soil physicochemical properties and microbial communities that favor their own survival in the forest ecosystem [2]. Geographical conditions and soil properties directly or indirectly affect the structure and diversity of fungal communities [10]. As geographical distance increases, the similarity of soil fungal communities decreases [11]. We speculate that the symbiosis of *R. griseocarnosa* and host plants have an effect on the diversity and community structure of mycosphere soil. Hence, the present study examined the fungal community structure and environmental factors of the mycosphere and bulk soil of *R. griseocarnosa* originating from distinct geographical sites, and explored the effects of these different geographical sites on soil fungal communities. The purpose of this study was threefold: (1) to identify the biomarker genera of soil fungal communities in mycosphere soil from different geographical sites; (2) to clarify the effect of symbiosis of *R. griseocarnosa* and host plants on the diversity and community structure of mycosphere soil fungi; and (3) to understand the factors shaping the distribution of fungi in the mycosphere soil of *R. griseocarnosa*. Altogether, the results provide an empirical basis for the conservation of this valuable fungus in the wild, and the promotion of its propagation.

## 2. Materials and Methods

### 2.1. Sample Collection

Mycosphere and bulk soils were collected from five regions of Fujian, Guangxi, and Yunnan Provinces in China during the growing season of *R. griseocarnosa* in early July (Table 1). Host plants of *R. griseocarnosa* were also collected for identification, and the temperature range, latitude, longitude, and elevation of each location also recorded. Their temperature range was 24–37 °C, and the soil types at different sampling locations were ferralsols. For the collection of mycosphere soil, a spiral drill was used to collect soil attached to the roots of *R. griseocarnosa* at a depth of 0–10 cm, which was then placed into sterile self-sealing bags [12]. The lateral distance between each *R. griseocarnosa* fruiting body was at least 1 m [12]. Additionally, bulk soil from nearby areas without *R. griseocarnosa* was collected at a depth of 0–10 cm, maintaining a lateral distance of 40 cm from each *R. griseocarnosa*, and placed into sterile self-sealing bags [12]. Across five sites, three mycosphere soil samples were collected at the DPF site, while three mycosphere soil samples and corresponding bulk soil samples were collected at each sampling point in the other four sites, totaling 27 samples. We then divided the collected soil samples into two parts, and one part was air-dried naturally and sieved through a 2 mm sieve for the determination of soil physical and chemical properties, while the other part was stored in a –80 °C in freezer for molecular analysis.

### 2.2. Analysis of Soil Physical and Chemical Properties

The physical and chemical properties of the soil samples after air-drying each were analyzed, and all the measurements were calculated on a dry weight basis. Soil pH was recorded with an acidity meter (EutechCyberScan pH510, Eutech Instruments, Vernon Hills, IL, USA) using a soil-to-water ratio (*w*/*v*) of 1:2.5 [13]. Soil organic carbon (SOC) was measured by the potassium dichromate heating method [14]. To determine the soil available phosphorus (AP), molybdenum antimony resistance colorimetry with double acid extraction was used [15]. Soil available potassium (AK) was determined by flame spectrophotometry [16], while soil available nitrogen (AN) was determined by the alkaline hydrolysis diffusion method [17].

### 2.3. Analysis of Soil Fungal Diversity and Community Structure

From each soil sample, 0.30 g was weighed to extract its DNA, by following the instructions of the Ezup column soil DNA extraction kit (B518233, Sangon Biotech (Shanghai) Co., Shanghai, China). The DNA concentration and purity were assessed on a NanoDrop 2000 spectrophotometer, to ensure that the former was >50 mg/L and the latter’s OD_260/280_ ratio was between 1.8 and 2.0. The qualified DNA samples were then sequenced for fungal ITS diversity. The primer fragment size for ITS sequencing was approximately 300 bp, and the primers were ITS1F (5′-CTTGGTCATTTAGAGGAAGTAA-3′) and ITS2R (5′-GCTGCGTTCTTCATCGATGC-3′) [18]. The PCR reaction system consisted of the following: 5× amplification buffer (4 μL), 2.5 mmol/L dNTPs (2 μL), 2.0 g/L bovine serum albumin solution (BSA, 0.2 μL), 5 μM forward (0.8 μL) and reverse primers (0.8 μL), 0.5 U/μL Taq DNA polymerase (0.4 μL), 50 mg/L DNA template (2 μL), ultra-pure water (9.8 μL). The PCR reaction procedure was as follows: predenaturation at 95 °C for 3 min, followed by 35 cycles at 95 °C for 10 s, 55 °C for 30 s, and 72 °C for 45 s, then ending with a final extension at 72 °C for 10 min (GeneAmp 9700 thermocycler PCR system, Applied Biosystems, Beijing, China). The PCR products were detected by agarose gel electrophoresis with a mass to volume ratio of 2%, and then sequenced (2 × 300 bp) using Illumina’s MiSeq platform (Majorbio Bio-Pharm Technology Co., Shanghai, China). The generated raw data have been stored in the NCBI Sequence Read Archive (SRA) database (accession number: PRJNA1212833).

The raw fastq was demultiplexed, and quality-filtered by fastp v 0.19.6, and then merged by FLASH v 1.2.7 using the following criteria: (1) truncated 300 bp reads with an average quality score <20 (over 50 bp); any truncated reads <50 bp were discarded; (2) precise barcode matching sequences were included, and any reads containing two nucleotide mismatches or ambiguous characters in primer matching were deleted; (3) those overlapping sequences exceeding 10 bp were assembled according to where they overlapped; and (4) all unassembled reads were discarded.

Next, the clean reads were clustered into operational taxonomic units (OTUs) with a 97% similarity cutoff value using USEARCH1 v7.0. The Ribosomal Database Project (RDP) classifier v 2.2 was used to analyzed OTUs based on the UNITE (Release 8.0) taxonomy database, with a 70% confidence threshold. Before performing the soil fungus calculations, sequences of *R. griseocarnosa* were removed to eliminate any estimation bias of *R. griseocarnosa* abundance [19]. Sequencing analysis was performed by smoothing the minimum number of sequences in the sample [20]. The diversity indices (Chao richness index, Shannon diversity index) were calculated using Mothur v.1.30.1, based on the OTU tables obtained for the samples. The fungal function was predicted by the FunGuild classification predicts database (Fungi Functional Guild, Guilds_v1.1.py), with the identified fungal OTUs assigned to nutritional groups and guilds via BLAST v 2.12.0+ search [21].

### 2.4. Statistical Analysis

Statistical analysis was performed using Welch’s t-test for unequal variances between two groups of data [22]. The fungal diversity indexes are presented as the mean ± standard error (SE). For multiple comparisons, the Kruskal–Wallis H test was implemented in STAMP v2.1.3. The relative abundance of fungal OTU was analyzed by detrended correspondence analysis (DCA), which showed that the length of the first axis was greater than 3 [23]. Therefore, based on the relative abundance of fungal species at OTU level, canonical correspondence analysis (CCA) was performed to reveal the association between fungal communities and environmental factors, using the vegan package in the R v4.1.3 software platform [23]. To analyze the relationships between mycosphere fungal communities and environmental factors, variation partitioning analysis (VPA) was also performed using R’s vegan package [24]. The distance–decay relationship was calculated as the ordinary least squares regression slope of the relationship between geographic distance and fungal community similarity (i.e., 1–dissimilarity measured by Bray Curtis) [25]. LEfSe (linear discriminant analysis (LDA) effect size) was used to discover the significant differences of five regions’ mycosphere fungal taxa at the genus level, under default parameters (LDA score > 2, *p* < 0.05). This was carried out online using the Majorbio I-Sanger Cloud Platform2 (https://cloud.majorbio.com/page/tools/, accessed on 11 October 2024). Spearman correlation coefficients between the LEfSe-significant fungal genera in the mycosphere and environmental factors were calculated and displayed as a heatmap using the pheatmap package in R v4.1.3 [26].

## 3. Results

### 3.1. Sample Site Information of Mycosphere and Bulk Soils

Compared with bulk soil, the SOC of HTCF (*p* = 0.009) was significantly higher in mycosphere soil, but vice versa for ZPF (*p* = 0.009) (Table 1). The AN, AP, and AK values were higher in all mycosphere soils compared with their bulk soils (Table 1). These results showed that relative to bulk soil, mycosphere soil was more nutrient-rich.

The mycosphere’s SOC content ranged from 3.49 to 11.74 g/kg, with a soil pH value between 3.97 and 4.50, and AN content of 198.26–378.08 mg/kg, an AP content of 3.87–18.48 mg/kg, and AK content of 106.21–345.57 mg/kg (Table 1). There were significant differences in soil physicochemical properties among soil samples from different mycosphere soils.

### 3.2. Soil Fungal Diversity of R. griseocarnosa

After flattening the minimum sequence for each sample, we obtained 39,600 sequences on average, and the rarefaction curve for the number of OTUs actually represented fungi tended to plateau (Figure S1). This demonstrated that the sequencing results were reasonable and reliable, in that with more sequencing data found, the contribution rate of each new OTU was lower.

**Figure 1:**
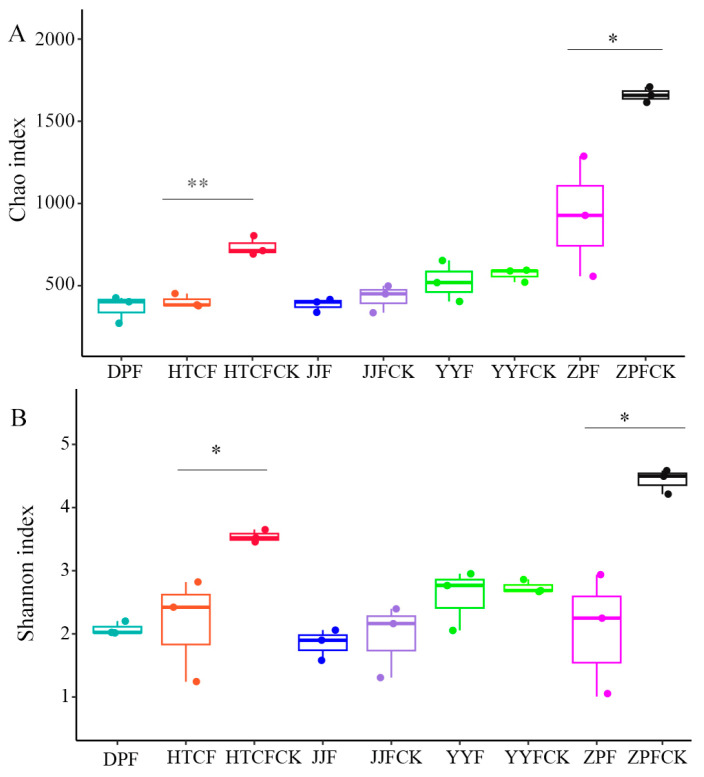
Comparison of Chao (**A**) and Shannon (**B**) diversity indexes between mycosphere and bulk soil. Significant differences by * *p* < 0.05; ** *p* < 0.01. DPF, HTCF, JJF, YYF, and ZPF represent mycosphere soil in different geographical areas, and HTCFCK, JJFCK, YYFCK, and ZPCK represent bulk soil.

We investigated the distinctiveness of fungal communities in the mycosphere and bulk soils sampled from the different sites. In terms of fungal diversity, the Chao (Figure 1A) and Shannon (Figure 1B) indexes of mycosphere soil samples from both HTCF and ZPF were significantly lower than those of their bulk soil samples. The Chao index of the five regions differed significantly (*p* = 0.016), among which ZPF’s Chao diversity index was the highest (Figure S2).

### 3.3. Soil Fungal Community Structure of R. griseocarnosa

**Figure 2:**
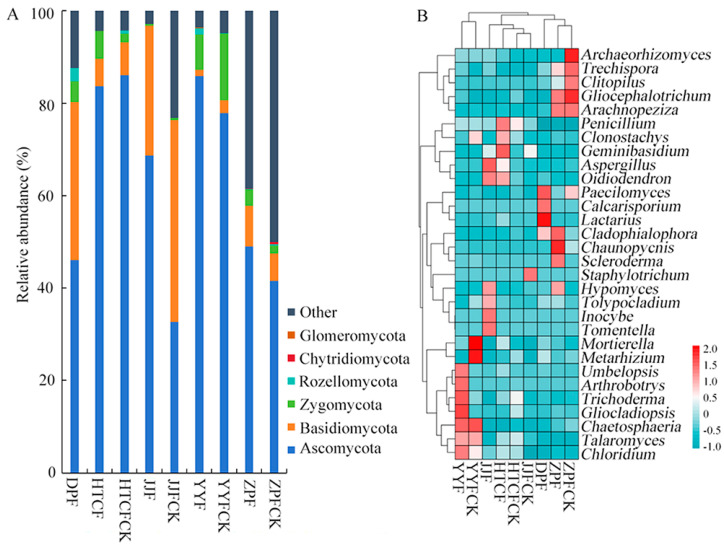
Comparison of fungi community composition between mycosphere and bulk soil. (**A**) Phylum level; (**B**) the top 30 genera. DPF, HTCF, JJF, YYF, and ZPF represent mycosphere soil in different geographical areas, and HTCFCK, JJFCK, YYFCK, and ZPCK represent bulk soil.

We clustered the obtained OTUs into six phyla. Among all soil samples, Ascomycota, Basidiomycota, and Zygomycota were the main phyla found (Figure 2A). Overall, we identified 411 genera in the sequencing dataset, with *Penicillium*, *Trichoderma*, *Talaromyces*, *Mortierella*, and *Tolypocladium* being the dominant genera. We observed that *Penicillium*, *Tolypocladium*, *Chloridium*, *Oidiodendron*, and *Umbelopsis* levels were higher in mycosphere soil than bulk soil at all sites (Figure 2B). In the HTCF area, the abundance of *Mortierella* (*p* = 0.031) was significantly higher in its mycosphere soil relative to its bulk soil (Figure 2B), whereas that of *Chaetosphaeria* (*p* = 0.046) was significantly lower in mycosphere soil (Figure 2B). Conversely, *Mortierella* was significantly higher in the bulk soil than mycosphere soil at YYF (*p* = 0.023) (Figure 2B). *Paecilomyces* was significantly higher in the bulk soil of ZPF relative to its mycosphere soil (*p* = 0.0022) (Figure 2B).

**Figure 3:**
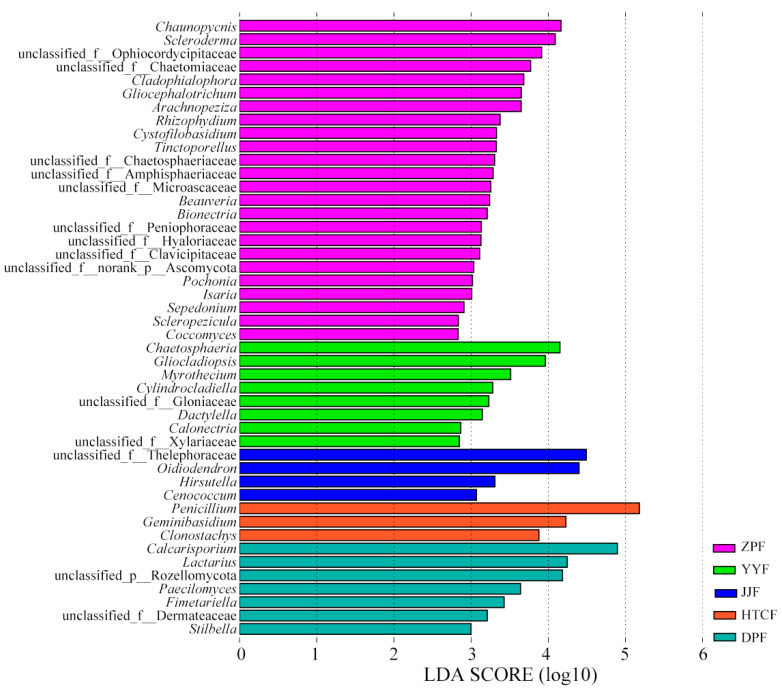
LEfSe analysis of mycosphere fungi at genus level. DPF, HTCF, JJF, YYF, and ZPF represent mycosphere soil in different geographical areas.

The LEfSe (linear discriminant analysis (LDA) effect size) analysis of the fungal communities from five regions revealed significant bioindicator fungal genera in the mycosphere soils (LDA score > 2, *p* < 0.05). There were 46 LEfSe-significant fungal genera (Figure 3). The fungal biomarker genera at ZPF comprised *Chaunopycnis*, *Scleroderma*, *Cladophialophora*, *Gliocephalotrichum*, and *Arachnopeziza*, while those at YYF consisted of *Chaetosphaeria* and *Gliocladiopsis*. Those at JJF were limited to only *Oidiodendron*. Those at HTCF were *Penicillium*, *Geminibasidium*, and *Clonostachys*. Those at DPF were *Calcarisporium*, *Lactarius*, and *Paecilomyces* (Figure 3).

### 3.4. Effects of Abiotic and Biotic Factors on Soil Fungal Community Structure

**Figure 4:**
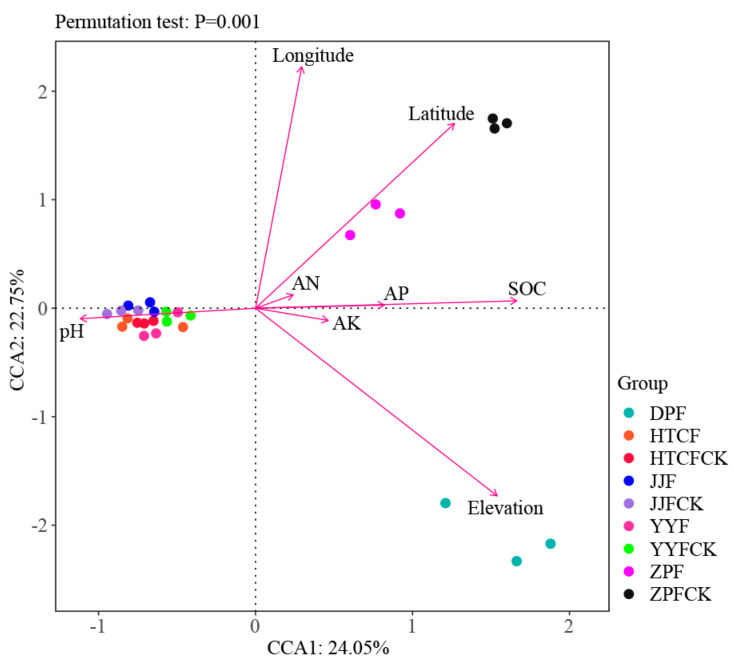
Canonical correspondence analysis (CCA) based on the relative abundance of fungal taxa at the OTU level and environmental factors. DPF, HTCF, JJF, YYF, and ZPF represent mycosphere soil in different geographical areas, and HTCFCK, JJFCK, YYFCK, and ZPCK represent bulk soil. AK, AN, AP, and SOC represent available potassium, available nitrogen, available phosphorus, and soil organic carbon, respectively.

The CCA results describe the relation between environmental factors and soil fungi microbial community structure (Figure 4). They showed that the CCA1 and CCA2 axes explained 24.05% and 22.75% of the total community variance, respectively (Figure 4). The results also showed that pH (r^2^ = 0.468, *p* = 0.0015), SOC (r^2^ = 0.695, *p* < 0.001), AP (r^2^ = 0.343, *p* = 0.014), elevation (r^2^ = 0.967, *p* < 0.001), longitude (r^2^ = 0.937, *p* < 0.001), and latitude (r^2^ = 0.886, *p* < 0.001) were the main factors influencing the compositional assembly of soil fungal communities (Figure 4). In particular, the samples from HTCF, JJF, and YYF were predominantly affected by pH, whereas those from DPF were influenced by elevation, and those from ZPF and ZPFCK were influenced by latitude (Figure 4).

**Figure 5:**
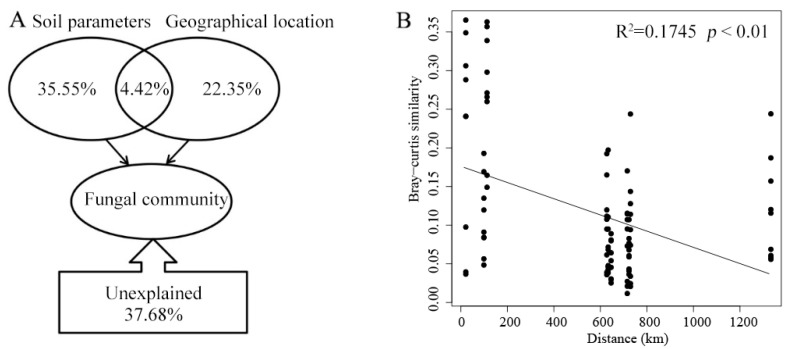
Relationships between mycosphere fungal communities and environmental factors. (**A**) Variation partition analysis (VPA) of soil/site properties on fungal community. (**B**) Distance–decay curves of fungal communities.

To quantify the effects of soil parameters and geographical location (longitude, latitude, and elevation) on mycosphere fungal communities, we used variance partitioning analysis (VPA). These variables explained a substantial portion (62.32%) of the changes in fungal community structure in the mycosphere (Figure 5A). Notably, of that percentage, the soil parameters constituted 35.55% and geographical location accounted for 22.35%, while their interactions explained just another 4.42% of the variation among mycosphere fungal communities (Figure 5A). The beta diversity (Bray–Curtis dissimilarity) of the mycosphere fungal community decreased with an increase in geographical distance (Figure 5B).

**Figure 6:**
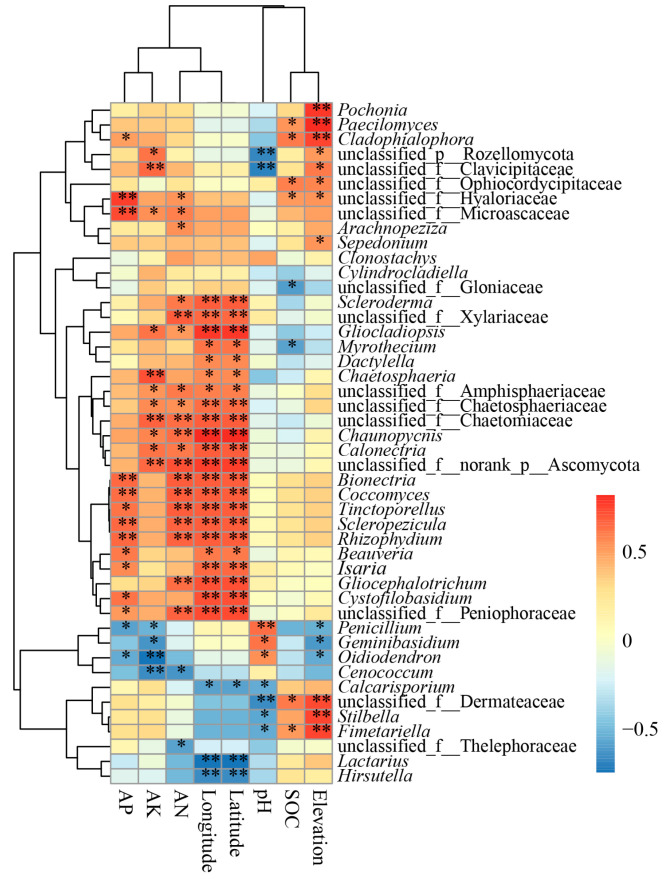
Spearman correlation between the LEfSe-significant fungal genera and soil/site properties. Significant correlation was noted when * *p* < 0.05 and ** *p* < 0.01.

The relative abundance of the LEfSe-significant fungal genera and soil/site properties were analyzed by Spearman correlations in the mycosphere soil (Figure 6). The resulting heatmap showed that longitude, latitude, and AN clustered together; AP and AK gathered together; and elevation and SOC aggregated together, but pH was set largely apart and clustered alone (Figure 6). *Penicillium*, *Geminibasidium*, and *Oidiodendron* were negatively correlated with AP (*p* < 0.05), AK (*p* < 0.05), and elevation (*p* < 0.05), and positively correlated with pH (*p* < 0.05) (Figure 5). *Calcarisporium* was negatively correlated with pH (*p* = 0.04), longitude (*p* = 0.024), and latitude (*p* = 0.024) (Figure 6). *Chaunopycnis* and *Calonectria* showed positive correlation with AK, AN, longitude, and latitude (*p* < 0.05) (Figure 6). *Bionectria*, *Coccomyces*, *Tinctoporellus*, *Scleropezicula*, and *Rhizophydium* showed positive correlation with AP, AN, longitude, and latitude (*p* < 0.05) (Figure 6). *Paecilomyces*, *Cladophialophora*, and *Fimetariella* showed a significant positive correlation with elevation (*p* < 0.01) and SOC (*p* < 0.05) (Figure 6). *Stilbella* and *Fimetariella* showed a significant positive correlation with elevation (*p* < 0.01), while being negatively correlated with pH (*p* < 0.05). *Lactarius* and *Hirsutella* had significant negative correlations with longitude and latitude (*p* < 0.01) (Figure 6).

### 3.5. Functional Predictions of Soil Fungi

We assigned fungal OTUs to specific nutrient groups and then further subdivided them into specific roles in ecology. Three ecological functions were mainly detected, namely pathotroph, saprotroph, and symbiotroph (Table S1). Pathotroph–symbiotroph (ericoid mycorrhizal) and saprotroph–symbiotroph (ectomycorrhizal lichenized wood saprotroph) were more common in mycosphere soil than bulk soil at all sites (Table S1). At the JJF and YYF sites, saprotroph (undefined saprotroph and soil saprotroph) and saprotroph–symbiotroph (ectomycorrhizal-undefined saprotroph) were more prevalent in mycosphere soil than bulk soil (Table S1). At HTCF, pathotroph–saprotroph (animal pathogen-fungal parasite-undefined saprotroph), pathotroph–symbiotroph (ericoid mycorrhizal), and saprotroph–symbiotroph (endophyte-litter saprotroph-soil saprotroph-undefined saprotroph) were greater in mycosphere soil than bulk soil (Table S1). In the ZPF samples of mycosphere soil, pathotroph (fungal parasite), pathotroph–symbiotroph (animal pathogen-clavicipitaceous endophyte-fungal parasite), saprotroph (undefined saprotroph), and symbiotroph (ectomycorrhizal) were all higher than in bulk soil (Table S1).

**Figure 7:**
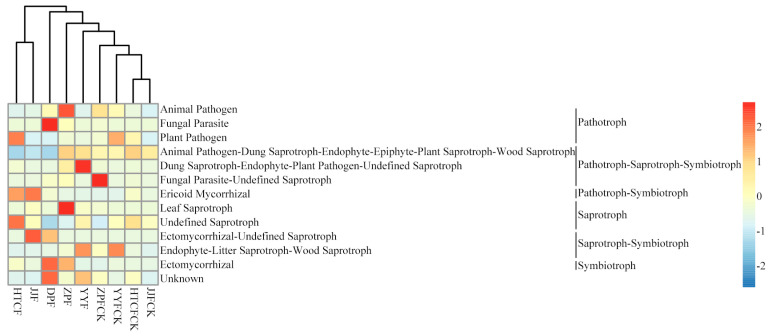
Functional classification of the LEfSe-significant fungal genera. DPF, HTCF, JJF, YYF, and ZPF represent mycosphere soil in different geographical areas, and HTCFCK, JJFCK, YYFCK, and ZPCK represent bulk soil.

The functional analysis of LEfSe-significant fungal genera showed that fungal parasite and ectomycorrhizal abundance at DPF significantly exceeded that of other mycosphere site samples (Figure 7). Yet significant geographic differences in saprotroph (leaf saprotroph) abundance were evident, being significantly higher in the samples of ZPF than those of other sites (*p* = 0.027) (Figure 7). The abundance of dung saprotroph-endophyte-plant pathogen-undefined saprotroph at YYF was significantly higher than that of other site samples (*p* = 0.040). Finally, the abundance of undefined saprotroph at HTCF significantly exceeded that of other site samples (*p* = 0.019) (Figure 7).

## 4. Discussion

Consistent with most studies concerning soil fungal communities [12,19,27], we found that fungal Chao and Shannon diversity indices in mycosphere soil were lower than in bulk soil. This indicates that the symbiosis of *R. griseocarnosa* and host plants has a significant impact on the diversity and community structure of soil mycosphere. Among the mycosphere soil fungi, the reduction in Basidiomycota (Figure 2A) may be explained by their rejection by *R. griseocarnosa*, since the latter secretes antifungal compounds that repel other basidiomycetes, thus promoting its own growth [28,29]. This belowground reduction of Basidiomycota also occurs with other ectomycorrhizal fungi [18], leading to a decline in the diversity of soil fungi [19]. Furthermore, Yu et al. [4] found that the richness and diversity of mycorrhizal soil fungi decreased during the symbiosis stage in *Russula*–Fagaceae root interactions. According to Yu et al. [4], the abundance of *Russula*’s DNA features in mycosphere soil increased in the stage of mycelium growth. The sequences of *R. griseocarnosa* dominated the sequencing data and obscured the signals of other low-abundance fungi, leading to bias in soil fungal community structure analysis. After eliminating the sequences of *R. griseocarnosa*, rare species or key functional bacteria can be identified more clearly. To better understand the relationship between *R. griseocarnosa* and soil fungi, we deleted the *R. griseocarnosa* sequence to eliminate bias in estimating fungal community structure and diversity [19].

The mycosphere fungal community of *R. griseocarnosa* was mainly composed of Ascomycota, Basidiomycota, and Zygomycota (Figure 2A). The majority of Ascomycota are saprophytic fungi, which can decompose recalcitrant substances such as lignin and keratins, thereby bolstering nutrients’ circulation in soil and improving overall soil quality [30]. Frey et al. [31] found that Ascomycota and Zygomycota fungi are critically involved in the decomposition of plant residues and organic matter in soil, while Basidiomycota members have a strong ability to decompose lignocellulose. Oh et al. [18] reported that the main fungi in the mycosphere soil of the edible fungus *Tricholoma matsutake* belonged to Ascomycota, and likewise for the mycosphere soil of *Russula* as well as *Craterellus* [32]. In mycosphere soil, the dominant fungi were *Penicillium*, *Trichoderma*, *Talaromyces*, *Mortierella*, and *Tolypocladium* (Figure 2B). Most studies have shown that *Penicillium* spp. are the dominant genus in the mycosphere soil of mycorrhizal edible fungi, whose presence and activity may benefit the fruit body development of mycorrhizal species [33,34]. *Trichoderma* spp. can secrete cellulase and hemicellulase to degrade plant cell walls [35] and dissolve organic and inorganic phosphorus in soil, thereby promoting the absorption of soil phosphorus by plants and participating in the soil carbon and phosphorus cycle [36]. Work by Sun et al. [37] found that the saprophytic *Mortierella* fungi occur at high abundance in soils rich in organic matter and are major microbial members responsible for soil organic matter and nutrient cycling. Yu et al. [4] detected *Mortierella*, *Penicillium*, and *Trichoderma* in the mycorrhizal mycosphere during both the *Russula*-Fagaceae root mycelia-running stage and the *Russula* sporocarping stage. Our results showed that both *Tomentella* and *Umbelopsis* were more abundant in mycosphere soil than bulk soil (Figure 2B). Other research has shown that *Tomentella* can form ectomycorrhizae improving the absorption and accumulation of mineral nutrients in soil by the host plant [38], while *Umbelopsis* has been detected often in the fruiting bodies and fairy rings of *Tricholoma matsutake* mushrooms [39]. Oh et al. [19] had suggested that *Umbelopsis* and *Mortierella* could interact positively with *T. matsutake*, which is consistent with our results. According to Yu et al. [4], the existence of *Tomentella* and *Chaetosphaeria* may indicate a shift in *Russula*’s physiological development, from vegetative (mycelium) stage to sporocarp production (reproductive) phase. A study by Qi et al. [27] demonstrated that *Trichoderma*, *Inocybe*, and *Penicillium* were the main mycosphere fungi of *Russula* spp. Therefore, mutualistic fungi may coexist in mycelium-dominated soil habitats.

The growth of ectomycorrhizal fungi is influenced by biotic and abiotic factors in soil ecosystems [40]. Here, the contents of AN, AK, and AP were higher in mycosphere soil than in bulk soil (Table 1), and the CCA results showed that pH, SOC, AP, elevation, longitude, and latitude were the main factors influencing the composition of fungal communities. Yet soil pH emerged as the major factor influencing the structure of mycospheric fungal communities (Figure 4 and Figure 6). Studies have shown that *Boletus*, *Amanita*, and *Russula* ectomycorrhizal fungi often grow in forest habitats with a much thicker humus layer and fertile soil, with acidic soil of pH 4–6 being the most suitable for their growth [41,42]. The SOC content of mycosphere soil at HTCF and YYF was lower than that of the corresponding bulk soil, suggesting that *R. griseocarnosa* may regulate the soil fungal community, accelerate carbon decomposition, and thus reduce the organic carbon storage [43]. The DPF and ZPF samples were taken from high elevations, and the content of AP in those soil samples surpassed that in the other site samples (Table 1). As the elevation increased, the nutrient status of the mycosphere soil of dominant plants gradually became more phosphorus-limited [10]. We found that the main taxa of soil fungi with different geographical sites were similar (Figure 2B), but the biomarker genera occurred in these different geographical locations were different (Figure 3). That is, *Paecilomyces*, *Cladophialophora*, *Stilbella*, and *Fimetariella* were significantly positively correlated with SOC and elevation (Figure 6). Most genera were significantly positively correlated with longitude and latitude, while *Calcarisporium*, *Lactarius*, and *Hirsutella* were significantly negatively correlated with longitude and latitude (Figure 6). With increasing geographical distance, the community similarity of soil fungi decreased (Figure 5B), which is consistent with Yang’s findings [10]. Soil fungi exhibit strong local patterns that vary between different areas and which are driven by climate and soil factors [11]. These findings indicated that ectomycorrhizal fungi can affect the nutrient status of soil, and in turn, soil nutrients can regulate the colonization and growth of ectomycorrhizal fungi.

The soil fungi in the habitat of *R. griseocarnosa* were mainly saprophytic (Figure 7). Ectomycorrhizal fungi lack the GH6 and GH7 family genes encoding cellobiohydrolase, which are found in soil saprophytes [44]. This study found that the fertility of mycosphere soil was higher than that of bulk soil (Table 1), and the pathotroph–symbiotroph and saprotroph–symbiotroph functions of mycosphere were higher than those of bulk soil (Table S1). Mucha et al. [45] found that several saprophytic fungi could induce ectomycorrhizal fungi to produce chitinase and acid protease. High soil fertility leads to a general increase in the abundance of saprophytic and/or pathogenic fungi in forest ecosystems [46,47]. Since saprotrophic fungi can colonize in large numbers, they may continue to provide nutrients for the growth of *R. griseocarnosa* by helping the degradation of humus in the surrounding habitat [48,49,50]. Despite some limitations to functional predictions, we have uncovered potential positive effects of soil fungi on the growth of *R. griseocarnosa*, providing a sound basis for future studies to further explore the dynamic relationship between *R. griseocarnosa*’s growth dynamics and its soil microorganisms.

## 5. Conclusions

Through a comprehensive analysis of the physicochemical properties and fungal community structure of mycosphere of *R. griseocarnosa* from different geographical areas, it was found that soil AK, AN, and AP values were higher than those in bulk soil. *Penicillium*, *Trichoderma*, *Talaromyces*, *Mortierella*, *Tolypocladium*, *Chloridium*, *Oidiodendron*, and *Umbelopsis* were the dominant soil fungal taxa, which may promote the growth of *R. griseocarnosa*. There were pronounced differences in the indicator species from different geographical areas. However, the similarity of fungal communities in the mycosphere declined with the increase of geographical distance. Improved soil properties, including the application of nitrogen, phosphorus, potassium, and microbial fertilizers, could promote the propagation of *R. griseocarnosa*.

## Figures and Tables

**Table 1:** Sample site information of soil.

Sample	Soil Source	Location	Host Plant	Longitude (E)	Latitude (N)	Elevation (m)	pH	AK (mg/kg)	AN (mg/kg)	AP (mg/kg)	SOC (g/kg)
DPF	Mycosphere soil	Daping To., Yunnan Prov.	*Castanopsis chinensis*	104°35′23.85″	23°6′22.49″	1617	3.97 ± 0.07	265.78 ± 17.68	247.60 ± 26.03	11.70 ± 1.98	11.74 ± 3.59
HTCF	Mycosphere soil	Huangtianchong, Guangxi Prov.	*Castanopsis chinensis*	110°41′59.24″	23°10′33.92″	149	4.50 ± 0.03	106.21 ± 3.89	286.12 ± 33.07	3.87 ± 0.46	6.69 ± 0.74
HTCFCK	Bulk soil						4.50 ± 0.12	71.70 ± 7.55	179.40 ± 25.11	1.67 ± 0.51	2.56 ± 0.46
JJF	Mycosphere soil	Jinji Town, Guangxi Prov.	*Psychotria asiatica*	110°49′18.61″	23°13′36.54″	38	4.18 ± 0.05	141.25 ± 12.88	198.26 ± 23.12	6.88 ± 1.38	3.64 ± 0.11
JJFCK	Bulk soil						4.30 ± 0.02	89.49 ± 20.72	172.50 ± 13.59	3.65 ± 0.65	4.63 ± 2.13
YYF	Mycosphere soil	Youyi Co., Guangxi Prov.	*Ardisia quinquegona*	111°33′35.09″	23°41′30.84″	43	4.13 ± 0.04	345.57 ± 31.06	295.32 ± 5.75	5.55 ± 0.18	3.49 ± 0.15
YYFCK	Bulk soil						4.22 ± 0.05	123.46 ± 21.66	246.56 ± 62.33	3.63 ± 1.35	2.61 ± 0.89
ZPF	Mycosphere soil	Zhangping Co., Fujian Prov.	*Toona ciliata*	117°25′11.99″	25°17′24.66″	168	4.17 ± 0.02	333.15 ± 9.47	378.08 ± 12.34	18.48 ± 3.09	7.15 ± 1.26
ZPFCK	Bulk soil						3.88 ± 0.07	154.93 ± 12.35	230.17 ± 18.33	6.31 ± 6.31	13.91 ± 0.68

AK, AN, AP, and SOC represent available potassium, available nitrogen, available phosphorus, and soil organic carbon, respectively. Data are mean ± standard error (n = 3).

## Data Availability

The raw fastq file was stored in the NCBI Sequence Read Archive (SRA) database (Accession No: PRJNA1212833).

## Supplementary Materials

The following supporting information can be downloaded at https://www.mdpi.com/article/10.3390/microorganisms13030490/s1, Figure S1. Rarefaction curves for fungal sequences. Figure S2. Comparison of Chao (A) and Shannon (B) indexes in mycosphere soil. Different lowercase letters indicate significant differences (*p* < 0.05). Table S1. The relative abundances of soil fungal functional guilds.

## References

[1] Wang X.H., Yang Z.L., Li Y.C., Knudsen H., Liu P.G. (2009). *Russula griseocarnosa* sp. nov. (Russulaceae, Russulales), a commercially important edible mushroom in tropical China: Mycorrhiza, phylogenetic position, and taxonomy. Nova Hedwig..

[2] Liu Y., Yong T., Cai M., Wu X., Guo H., Xie Y., Hu H., Wu Q. (2024). Exploring the Potential of *Russula griseocarnosa*, A Molecular Ecology Perspective. Agriculture.

[3] Chen X.H., Xia L.X., Zhou H.B., Qiu G.Z. (2010). Chemical Composition and Antioxidant Activities of *Russula griseocarnosa* sp. nov. J. Agric. Food Chem..

[4] Yu W.Y., Peng M.H., Wang J.J., Ye W.Y., Li Y.L., Zhang T., Wang A.R., Zhang D.M., Wang Z.H., Lu G.D. (2021). Microbial community associated with ectomycorrhizal *Russula* symbiosis and dominated nature areas in southern China. FEMS Microbiol. Lett..

[5] Bergmann J., Verbruggen E., Heinze J., Xiang D., Chen B., Joshi J., Rillig M.C. (2016). The interplay between soil structure, roots, and microbiota as a determinant of plant–soil feedback. Ecol. Evol..

[6] Horn S., Hempel S., Verbruggen E., Rillig M.C., Caruso T. (2017). Linking the community structure of arbuscular mycorrhizal fungi and plants, a story of inter-dependence?. ISME J..

[7] Kataoka R., Siddiqui Z.A., Kikuchi J., Ando M., Sriwati R., Nozaki A., Futai K. (2012). Detecting nonculturable bacteria in the active mycorrhizal zone of the pine mushroom *Tricholoma matsutake*. J. Microbiol..

[8] Shen A., Shen B., Liu L., Tan Y., Zeng L., Tan Z., Li J. (2023). Diversity and Network Relationship Construction of Soil Fungal Communities in *Lactarius hatsudake* Tanaka Orchard during Harvest. Microorganisms.

[9] Frey-Klett P., Burlinson P., Deveau A., Barret M., Tarkka M., Sarniguet A. (2011). Bacterial-fungal interactions: Hyphens between agricultural, clinical, environmental, and food microbiologists. Microbiol. Mol. Biol. Rev..

[10] Yang Y., Qiu K.Y., Xie Y.Z., Li X.C., Zhang S., Liu W.S., Huang Y.Y., Cui L.Y., Wang S.Y., Bao P.G. (2023). Geographical, climatic, and soil factors control the altitudinal pattern of rhizosphere microbial diversity and its driving effect on root zone soil multifunctionality in mountain ecosystems. Sci. Total Environ..

[11] Tedersoo L., Mikryukov V., Zizka A., Bahram M., Hagh-Doust N., Anslan S., Prylutskyi O., Delgado-Baquerizo M., Maestre F.T., Pärn J. (2022). Global patterns in endemicity and vulnerability of soil fungi. Glob. Change Biol..

[12] Warmink J.A., van Elsas J.D. (2008). Selection of bacterial populations in the mycosphere of *Laccaria proxima*, is type III secretion involved?. ISME J..

[13] Wu J., He Z.L., Wei W.X., O’Donnell A.G., Syers J.K. (2000). Quantifying microbial biomass phosphorus in acid soils. Biol. Fert. Soils.

[14] Nelson D.W., Sommers L.E. (1996). Total carbon, organic carbon, and organic matter. Methods of Soil Analysis: Part 3 Chemical Methods.

[15] Retamal-Salgado J., Hirzel J., Walter I., Matus I. (2017). Bioabsorption and bioaccumulation of cadmium in the straw and grain of Maize (*Zea mays* L.) in growing soils contaminated with cadmium in different environment. Int. J. Environ. Res. Public Health.

[16] Zhao J., Zhang R.F., Xue C., Xun W.B., Sun L., Xu Y.C., Shen Q.R. (2014). Pyrosequencing reveals contrasting soil bacterial diversity and community structure of two main winter wheat cropping systems in China. Microb. Ecol..

[17] Liu P., Wang X.H., Li J.G., Qin W., Xiao C.Z., Zhao X., Jiang H.X., Sui J.K., Sa R.B., Wang W.Y. (2015). Pyrosequencing reveals fungal communities in the rhizosphere of Xinjiang jujube. BioMed Res. Int..

[18] Adams R.I., Miletto M., Taylor J.W., Bruns T.D. (2013). Dispersal in microbes, fungi in indoor air are dominated by outdoor air and show dispersal limitation at short distances. ISME J..

[19] Oh S.Y., Fong J.J., Park M.S., Lim Y.W. (2016). Distinctive feature of microbial communities and bacterial functional profiles in *Tricholoma matsutake* dominant soil. PLoS ONE.

[20] Ye J., Joseph S.D., Ji M., Nielsen S., Mitchell D.R.G., Donne S., Horvat J., Wang J.L., Munroe P., Thomas T. (2017). Chemolithotrophic processes in the bacterial communities on the surface of mineral-enriched biochars. ISME J..

[21] Nguyen N.H., Song Z., Bates S.T., Branco S., Tedersoo L., Menke J., Schilling J.S., Kennedy P.G. (2016). FUNGuild, an open annotation tool for parsing fungal community datasets by ecological guild. Fungal Ecol..

[22] Delacre M., Lakens D., Leys C. (2017). Why psychologists should by default use welch’s t-test instead of student’s t-test. Int. Rev. Soc. Psychol..

[23] Lepš J., Šmilauer P. (2003). Multivariate Analysis of Ecological Data Using CANOCO.

[24] Oksanen J., Blanchet F., Friendly M., Kindt R., Legendre P., McGlinn D., Minchin P., O’Hara R., Simpson G., Solymos P. (2020). Vegan Community Ecology Package Version 2.5—7 November 2020.

[25] Fan M.C., Li J.J., Luan X.B., Yang L., Chen W.Q., Ma X., Yang Z., Shangguan Z.P. (2023). Biogeographical patterns of rhizosphere microbial communities in *Robinia pseudoacacia* forests along a north–south transect in the Loess Plateau. Geoderma.

[26] Yu F., Liang J.F., Song J., Wang S.K., Lu J.K. (2020). Bacterial Community Selection of *Russula griseocarnosa* Mycosphere Soil. Front. Microbiol..

[27] Qi L.L., Wu X.J., Li L.Y., Mo C.M., Lang N., Chen Z.N. (2022). Diversity Research of Soil Fungi under Fruiting Bodies of *Russula griseocarnosa* and Its Related Species. Chin. J. Trop. Crops.

[28] Takakura Y. (2015). *Tricholoma matsutake* fruit bodies secrete hydrogen peroxide as a potent inhibitor of fungal growth. Can. J. Microbiol..

[29] An G.H., Cho J.H., Kim O.T., Han J.G. (2021). Metagenomic Analysis of Bacterial and Fungal Communities Inhabiting Shiro Dominant Soils of Two Production Regions of *Tricholoma Matsutake*, S. Ito & S. Imai in Korea. Forests.

[30] Yelle D.J., Ralph J., Lu F.C., Hammel K.E. (2008). Evidence for cleavage of lignin by a brown rot basidiomycete. Environ. Microbiol..

[31] Frey S.D., Knorr M., Parrent J.L., Simpson R.T. (2004). Chronic nitrogen enrichment affects the structure and function of the soil microbial community in temperate hardwood and pine forests. For. Ecol. Manag..

[32] Kim M., Yoon H., You Y.H., Kim Y.E., Woo J.R., Seo Y., Lee G.M., Kim Y.J., Kong W.S., Kim J.G. (2013). Metagenomic analysis of fungal communities inhabiting the fairy ring zone of *Tricholoma matsutake*. J. Microbiol. Biotechn..

[33] Ye L., Fu Y., Zou J., Li X.L. (2018). Isolation and identification of endophytic fungi from three commercial truffles in Sichuan province. Chin. Agric. Sci. Bull..

[34] Toju H., Sato H. (2018). Root-Associated Fungi Shared Between Arbuscular Mycorrhizal and Ectomycorrhizal Conifers in a Temperate Forest. Front. Microbiol..

[35] Rosolen R.R., Horta M.A.C., de Azevedo P.H.C., da Silva C.C., Sforca D.A., Goldman G.H., de Souza A.P. (2023). Whole-genome sequencing and comparative genomic analysis of potential biotechnological strains of *Trichoderma harzianum*, *Trichoderma atroviride*, and *Trichoderma reesei*. Mol. Genet. Genom..

[36] Minchiotti M.C., Vargas L.I., Madoery R.R. (2021). Phospholipase A activity and the biocontrol potential of *Trichoderma harzianum* and *Trichoderma atroviride*. Biocontrol Sci. Techn..

[37] Sun R.B., Dsouza M., Gilbert J.A., Guo X.S., Wang D.Z., Guo Z.B., Ni Y.Y., Chu H.Y. (2016). Fungal community composition in soils subjected to long-term chemical fertilization is most influenced by the type of organic matter. Environ. Microbiol..

[38] Lin S.S., Sun X.W., Wang X.J., Dou C.Y., Li Y.Y., Luo Q.Y., Sun L., Jin L. (2013). Mycorrhizal studies and their application prospects in China. Acta Prataculturae Sin..

[39] Oh S.Y., Park M.S., Cho H.J., Lim Y.W. (2018). Diversity and effect of Trichoderma isolated from the roots of Pinus densiflora within the fairy ring of pine mushroom (*Tricholoma matsutake*). PLoS ONE.

[40] Trappe M.J., Cromack K., Caldwell B.A., Griffiths R.P., Trappe J.M. (2012). Diversity of Mat-forming fungi in relation to soil properties, disturbance, and forest ecotype at crater Lake National Park, Oregon, USA. Diversity.

[41] Avis P.G., McLaughlin D.J., Dentinger B.C., Reich P.B. (2003). Long-term increase in nitrogen supply alters above-and below-ground ectomycorrhizal communities and increases the dominance of *Russula* spp. in a temperate oak savanna. New Phytol..

[42] Kujawska M.B., Rudawska M., Wilgan R., Leski T. (2021). Similarities and differences among soil fungal assemblages in managed forests and formerly managed forest reserves. Forests.

[43] Liu M.H., Wei Y.Q., Lian L., Wei B., Bi Y.X., Liu N., Yang G.W., Zhang Y.J. (2023). Macrofungi promote SOC decomposition and weaken sequestration by modulating soil microbial function in temperate steppe. Sci. Total Environ..

[44] Martin F., Aerts A., Ahren D., Brun A., Danchin E.G.J., Duchaussoy F., Gibon J., Kohler A., Lindquist E., Pereda V. (2008). The genome of *Laccaria bicolor* provides insights into mycorrhizal symbiosis. Nature.

[45] Mucha J., Dahm H., Strzelczyk E., Werner A. (2006). Synthesis of enzymes connected with mycoparasitism by ectomycorrhizal fungi. Arch. Microbiol..

[46] Castaño C., Dejene T., Mediavilla O., Geml J., Oria-de-Rueda J.A., Martín-Pinto P. (2019). Changes in fungal diversity and composition along a chronosequence of Eucalyptus grandis plantations in Ethiopia. Fungal Ecol..

[47] Guo W., Wang C., Brunner I., Zhou Y., Tang Q., Wang J., Li M.H. (2024). Responses of soil fungi to long-term nitrogen-water interactions depend on fungal guilds in a mixed *Pinus koraiensisforest*. J. Geophys. Res. Biogeosci..

[48] Kramer S., Marhan S., Ruess L., Armbruster W., Butenschoen O., Haslwimmer H., Kuzyakov Y., Pausch J., Scheunemann N., Schoene J. (2012). Carbon flow into microbial and fungal biomass as a basis for the belowground food web of agroecosystems. Pedobiologia.

[49] van der Wal A., Geydan T.D., Kuyper T.W., de Boer W. (2013). A thready affair, linking fungal diversity and community dynamics to terrestrial decomposition processes. FEMS Microbiol. Rev..

[50] Schmidt R., Mitchell J., Scow K. (2019). Cover cropping and no-till increase diversity and symbiotroph, saprotroph ratios of soil fungal communities. Soil Biol. Biochem..

