# Supplementary figures and images for: Soil Fungal Diversity and Community Structure of Russula griseocarnosa from Different Sites
Source: Microorganisms. 2025 Feb 22;13(3):490. doi: 10.3390/microorganisms13030490 (PMC11945854; doi:10.3390/microorganisms13030490)

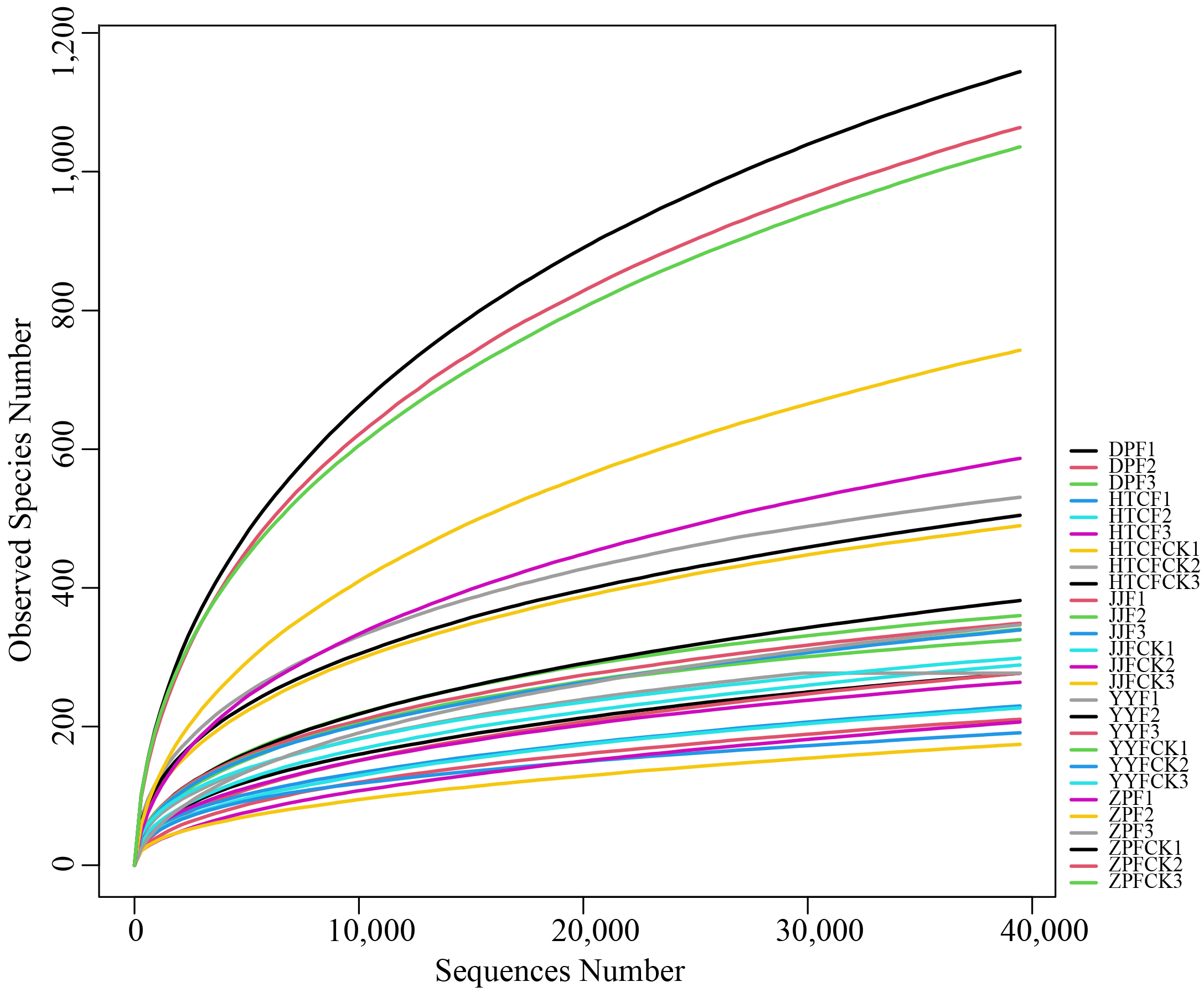

Supplement: Supplementary file 1 [file microorganisms-13-00490-s001.zip › Figure S1.jpg]

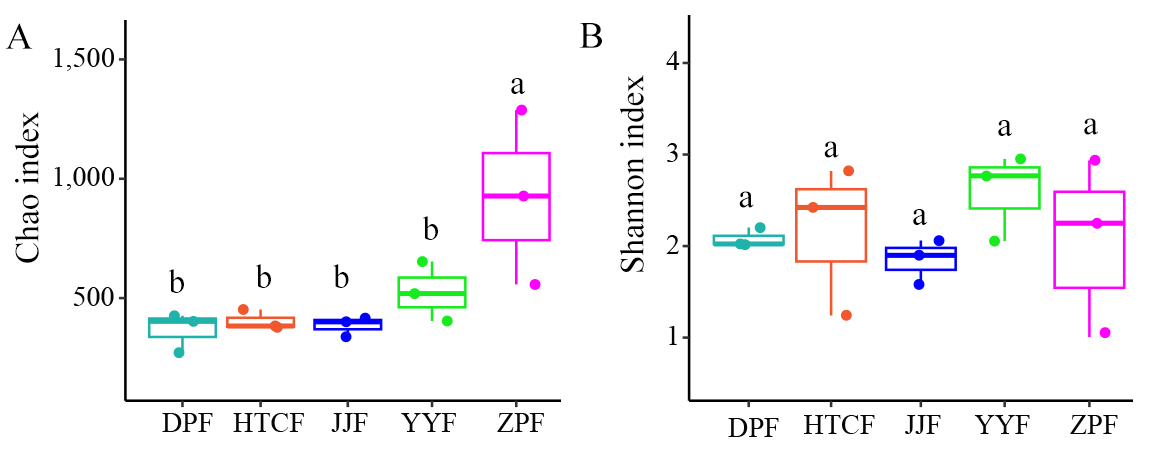

Supplement: Supplementary file 1 [file microorganisms-13-00490-s001.zip › Figure S2.jpg]
